# Trends in accessibility of negotiated targeted anti-cancer medicines in Nanjing, China: An interrupted time series analysis

**DOI:** 10.3389/fpubh.2022.942638

**Published:** 2022-07-22

**Authors:** Yanyan Liu, Huining Yi, Kexin Fang, Yuwen Bao, Xin Li

**Affiliations:** ^1^Nanjing Drum Tower Hospital, Clinical College of Nanjing Medical University, Nanjing, China; ^2^School of Health Policy and Management, Nanjing Medical University, Nanjing, China; ^3^Department of Regulatory Science and Pharmacoeconomics, School of Pharmacy, Nanjing Medical University, Nanjing, China; ^4^Center for Global Health, School of Public Health, Nanjing Medical University, Nanjing, China

**Keywords:** accessibility, pricing negotiation, targeted anti-cancer medicines, interrupted time series, policy intervention

## Abstract

**Background:**

In order to establish a long-term strategy for bearing the costs of anti-cancer drugs, the state had organized five rounds of national-level pricing negotiations and introduced the National Health Insurance Coverage (NHIC) policy since 2016. In addition, the National Healthcare Security Administration (NHSA) introduced the volume-based purchasing (VBP) pilot program to Nanjing in September 2019. Taking non-small cell lung cancer as an example, the aim of the study was to verify whether national pricing negotiations, the NHIC policy and the VBP pilot program had a positive impact on the accessibility of three targeted anti-cancer drugs.

**Methods:**

Based on the hospital procurement data, interrupted time series (ITS) design was used to analyze the effect of the health policy on the accessibility and affordability of gefitinib, bevacizumab and recombinant human endostatin from January 2013 to December 2020 in Nanjing, China.

**Results:**

The DDDs of the three drugs increased significantly after the policy implementation (*P* < 0.001, *P* < 0.001, *P* = 0.008). The trend of DDDc showed a significant decrease (*P* < 0.001, *P* < 0.001, *P* < 0.001). The mean availability of these drugs before the national pricing negotiation was <30% in the surveyed hospitals, and increased significantly to 60.33% after 2020 (*P* < 0.001, *P* = 0.001, *P* < 0.001). The affordability of these drugs has also increased every year after the implementation of the insurance coverage policy. The financial burden is higher for the rural patients compared with the urban patients, although the gap is narrowing.

**Conclusion:**

The accessibility of targeted anti-cancer drugs has increased significantly after the implementation of centralized prices, the NHIC policy and the VBP pilot program, and has shown sustained long-term growth. Multi-pronged supplementary measures and policy approaches by multiple stakeholders will facilitate equitable access to effective and affordable anti-cancer drugs.

## Background

### Current status of cancer

A globally aging population and rapid industrialization, along with risk factors such as chronic diseases, super bacteria, unhealthy lifestyles and environmental pollution, have markedly increased the global incidence of cancer worldwide. From 2006 to 2016 (1), the number of cancer patients increased by 38%, and the number of deaths increased by 17.8% across 195 countries and territories around the world, and cancer-related morbidity and mortality continue to rise (2, 3). The top 3 cancers in terms of incidence rates are lung cancer, stomach cancer and colorectal cancer, whereas lung cancer, liver cancer and stomach cancer rank foremost in terms of mortality rates (4). The accompanying surge in cancer treatment-related costs exerts a considerable burden on society and families (5, 6). Data from the National Cancer Center shows that in recent years, the annual cancer-related medical expenses in China have exceeded 220 billion yuan, and the out-of-pocket (OOP) expenses comprise more than half of the total family income (7, 8).

### Targeted anti-cancer drugs

In recent years, molecular targeted drugs and other innovative anti-cancer therapies have significantly prolonged the survival of cancer patients (9), alleviated pain and improved quality of life (10), thereby reducing the psychological pressure on family and community (11). However, due to the high costs of pharmaceutical research and development (12), patent protection of new drugs, limited national medical security capacity, skewed regional economic development, and differences in the diagnostic and treatment facilities of medical institutions, the availability of targeted anti-cancer drugs is severely restricted (13, 14). A famous Chinese movie named “Dying to Survive” was released in 2018, which described a story of cancer patients who could not afford the high cost of anticancer medicines and had to purchase illegal generic drugs from India (15).

### Counter-measures

In accordance with the principle of “government-led, policy linkage and continuous regulation,” the National Health Commission of China (NHC), in coordination with relevant departments, has adopted measures such as “group purchase” to exchange price for quantity, rational drug use, medical insurance payment, and R&D innovation on the basis of reducing the tax rate (16). The aim is to establish a long-term strategy to bear the costs of anti-cancer drugs. Since 2016, the state has organized the first round of national-level pricing negotiations and the National Health Insurance Coverage (NHIC) policy (17). In May 2016, the NHC announced the results of the first negotiation, and the prices of the targeted anti-cancer drugs icotinib and gefitinib used for the treatment of advanced non-small-cell lung cancer were reduced by 55% (18). In July 2017, the Ministry of Human Resources and Social Security of the People's Republic of China organized the second negotiation and introduced pharmacoeconomic evaluation as a negotiation tool for the first time (19). Eighteen drugs (including bevacizumab and recombinant human endostatin) were included in type B medicine list for national basic medical insurance, with an average price reduction of 44% (19). In October 2018, 17 drugs newly included in the medical insurance type B reimbursement catalog were announced in the third pricing negotiation (20), and their prices were reduced from 31 to 80%, thus greatly relieving the burden on patients (20). Ten innovative drugs that entered the Chinese market before 2018 were subjected to negotiations in November 2019 (21). New reforms were introduced by the NHIC in 2020 on account of the growing innovations in the development of anti-cancer drugs.

The centralization of the procurement of prescription drugs is being used in an increasing number of countries (22), and has the advantages of reducing drug prices, controlling drug expenditure, and improving drug accessibility by creating economies of scale (23). On 14th November 2018, the NHSA introduced the implementation of a new VBP pilot (i.e., the “4+7” pilot) program in 4 municipalities (Beijing, Shanghai, Tianjin, and Chongqing) and 7 sub-provincial cities (Guangzhou, Shenyang, Chengdu, Dalian, Xiamen, Xi'an, and Shenzhen) and the program was officially launched in March 2019 with the principle of combining tendering and procurement to achieve “volume-for-price” (24, 25). Since the pilot program was launched, the prices of 25 centrally purchased high-quality generic drugs including two targeted anti-cancer drugs, gefitinib and imatinib mesylate, have seen a significant reduction of 52% on average, with a maximum unit price reduction of 96% (24, 26). As for policy sustainability, four rounds of VBP have been successively implemented nationwide (27). In December 2019, the government announced that it would further expand the “4+7” pilot cities, and Nanjing was also included (28). Within 4 months of the launch of the pilot program in Nanjing, US$ 20 million was saved for the benefit of ordinary people, with a 59% reduction in the average price of 25 centrally purchased drugs (28).

### The effect of the NHIC policy

The NHIC policy was implemented in order to improve the accessibility and affordability of targeted anti-cancer drugs. Studies conducted outside China have shown that health insurance policies improve the willingness of patients to receive and continue treatment (29), reduce the economic burden (30, 31) and mortality rate of patients (32, 33), and increase the chances of receiving treatment by 25–35% (34). In addition, domestic studies have also confirmed that health insurance coverage increases the utilization of health services and lowers the economic burden of disease (16, 35). The current focus is on analyzing the change in the proportion of medical insurance and out-of-pocket payments, and the impact of lowered drug prices on the cost burden (36, 37). Diao et al. evaluated the impact of the provincial government health insurance program in Hangzhou and found that it improved the availability and affordability of 6 targeted anti-cancer drugs. Nevertheless, the financial burden remained high, especially for the rural low-income residents (38). Another group evaluated the price and availability of 15 innovative anti-cancer drugs included in the type B medicine list for national basic medical insurance in 2017 and found that the mean availability rate ranged from 27.44 to 47.33%, and the rate of price reduction was between 34 and 65% (16).

Nevertheless, it is challenging to introduce drugs covered by medical insurance into routine clinical practice due to the scope of drug reimbursement, the assessment of the proportion of drugs, the differences between the national and local insurance policies, and that between various hospitals. Therefore, it is critical to evaluate the implementation of the aforementioned policies to improve access to targeted anti-cancer drugs. Lung cancer is the most prevalent malignancy in China and is associated with high mortality rates (39). Gefitinib, bevacizumab and recombinant human endostatin are the first-line treatment drugs for non-small cell lung cancer (NSCLC) with somatic epidermal growth factor receptor (EGFR) mutations in China, and were included in the first and second batches of the price-negotiated anti-cancer drugs (40, 41). However, there is little evidence regarding the changes in the utilization, price, availability and affordability of these drugs for the treatment of NSCLC. The aim of the study was to verify whether the aforementioned policies have a positive impact on the accessibility of these targeted anti-cancer drugs in Nanjing, China.

## Methods

### Study design

The procurement of gefitinib, bevacizumab and recombinant human endostatin by various hospitals in Nanjing from January 2013 to December 2020 was analyzed using the interrupted time series (ITS) design. The primary reasons that we chose these three drugs included three aspects. First of all, lung cancer is the most prevalent malignancy in China and is associated with high mortality rates. And, gefitinib, bevacizumab and recombinant human endostatin are the first-line treatment drugs for NSCLC with somatic EGFR mutations in China. In the next place, these three drugs were included in the first and second batches of the price-negotiated anti-cancer drugs, and were approved earlier in China. This ensures sufficient observation period before and after the policy. The last reason, the data retrieved from the Nanjing Regional Hospital Drug Analysis System database is sufficient.

### Setting

Nanjing is located on the southeast coast of China, which is the capital of Jiangsu Province. In 2021, Nanjing has a population of over 9 million people in 11 municipal districts. The total gross domestic product (GDP) of Nanjing was 248.1 billion US$ (42), making it the city with the middle- and upper-level economic development area in Eastern China.

### Data source

The monthly purchasing data was retrieved from the Nanjing Regional Hospital Drug Analysis System database, which was jointly established with the support of Jiangsu Provincial Science and Technology Department and Provincial Health Commission. Totally, the 8 secondary hospitals and 23 tertiary hospitals in Nanjing were included in the study. The sampled hospitals were accounting for 24.24% of secondary hospitals and 82.14% of tertiary hospitals in Nanjing, respectively. Primary hospitals were excluded since they are not qualified to prescribe targeted anti-cancer drugs.

### Data cleaning and filtering

The Data Cleaning workflow is comprised of three steps. The initial basic action was to ingest the monthly sales data from the various data sources and identify the sampled hospitals. For the sake of improving the data quality and the analysis outcomes, the next step was filling the missing values. Afterwards, the last step was Data Verification. To ensure reliability, Data Verification was completed independently by two research assistants.

### Statistical analysis

#### Utilization

The monthly DDDs were calculated by dividing the monthly sales data in volume by DDD, defined as the daily amounts based on dosage regimen recommended in the manufacturer's instructions as approved by National Medical Products Administration (NMPA) (43). Higher DDDs indicated greater frequency of usage.

$$\begin{array}{l} DDDs\;=\;sales\;data\;in\;volume/DDD. \end{array}$$

#### Price

The daily cost of drugs was measured in terms of DDDc as below:

$$\begin{array}{l} DDDc\;=\;expenditures/DDDs. \end{array}$$

#### Availability

The availability of medicine was calculated as the percentage of the surveyed hospitals that stocked the drugs within the time period.

*Very low:* <*30%, hardly available in the surveyed hospitals*.
*Low: 30–49%, available in few hospitals*.
*Fairly high: 50–79%, available in many hospitals*.
*High:* ≥*80%, available in most hospitals*.

#### Affordability

As per the methodology of WHO/Health Action International (HAI), the affordability of each drug was calculated as the number of days' wages needed by the lowest-paid unskilled government worker to purchase a course of treatment based on standard treatment regimens. Treatment course requiring more than 1 day's wages is considered unaffordable (44). Data on the per capita annual disposable income from 2012 to 2020 was obtained from the Nanjing Statistical Yearbook. Given the long-term treatment and heavy financial burden of targeted anti-cancer drugs, the expenditure was also assessed in terms of the median progression-free survival (mPFS) that was evaluated based on the treatment guidelines.

*The OOP expenditure for the medicines per patient*=*the total cost of medicine* × *(1 – the proportion of reimbursement)*.

*Availability of patients* = *OOP expenditure for the medicine for achieving mPFS /per capita annual disposable income*.

Pharmaceutical-sponsored patient-assistance programs (PAPs) have been established to improve the access of low-income and uninsured patients to cancer drugs and decrease the economic burden. Patients who have purchased a prescribed course of treatment can apply for the free drug program through the hospital. Gefitinib and bevacizumab are included in the PAPs (Table 1). If the outcome of availability is <1, the drug is generally affordable for patients, and if the outcome of availability is >1, the drug is non-affordable for patients.

**Table 1:** Descriptive information and multiple interventions of gefitinib, bevacizumab, and recombinant human endostatin.

**Generic Name**	**Approval date in China**	**National pricing negotiations**	**Marketing Authorization Holder**	**Dosage Form**	**DDD (mg)**	**PAPs before pricing negotiation**	**The first policy intervention point**	**The second policy intervention point**
Gefitinib (branded drug)	2004	2016.7	AstraZeneca AB	Tablet	250	Free after payment of 8 months	2016.7	2020.1
Gefitinib (generic drug)	2017	—	Qilu	Tablet	250	—	—	2020.1 (the VBP pilot program)
Bevacizumab	2010	2017.9	Roche	Injection	25	Free after payment of 4 months	2017.9	2020.1 (renewal)
Recombinant human endostatin	2006	2017.9	Simcere	Injection	8.5	—	2017.9	2020.1 (renewal)

**Figure 1:**
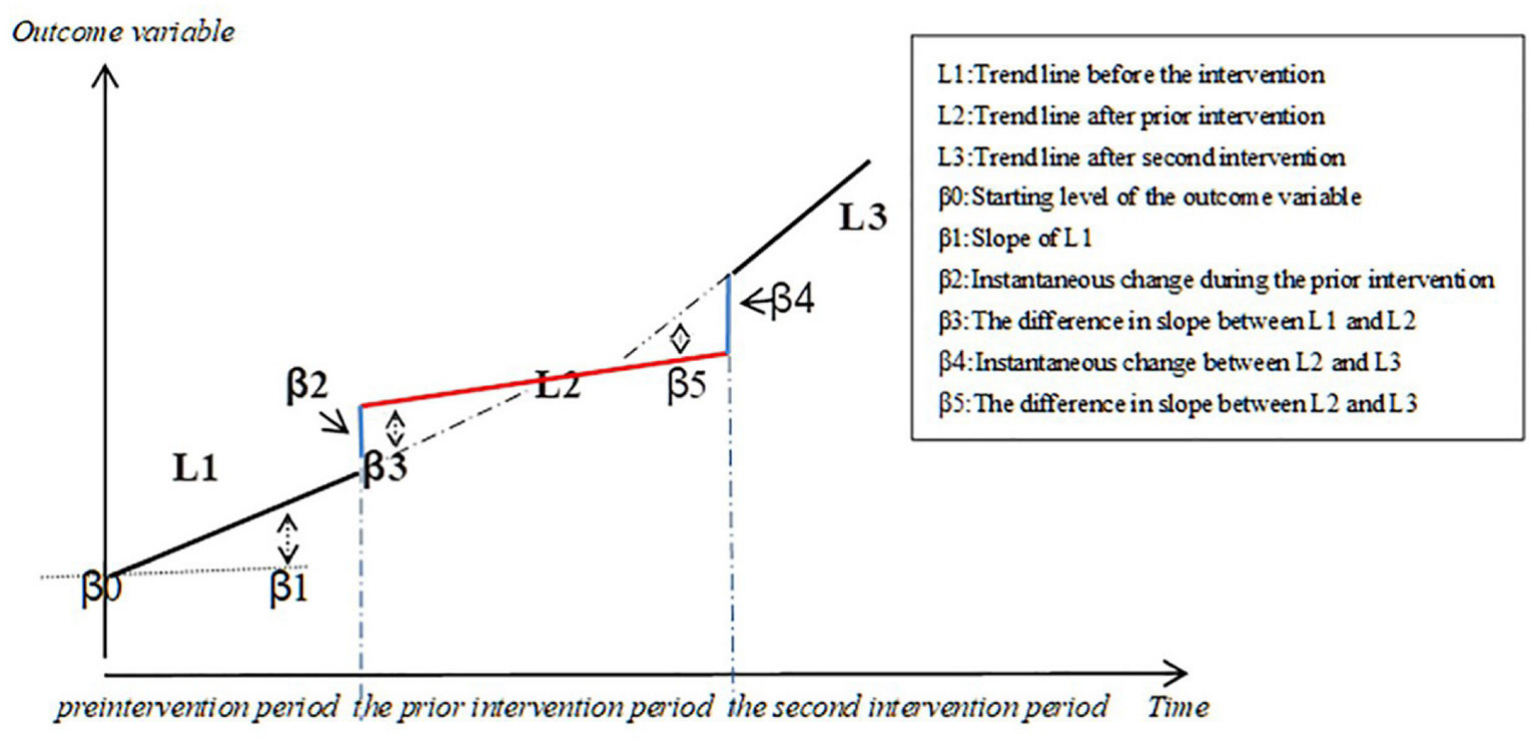
Graphic illustration of the ITS model and the trend lines for data points before and after policy implementation.

ITS regression analysis was used to evaluate the changes in the utilization of negotiated targeted anti-cancer drugs over a 96 month-period based on DDDs, DDDc, the level of availability, and affordability. The regression equation is as follows (Figure 1):

the single-treatment period analysis:

$$\begin{array}{l} Yt=\beta 0+\beta 1^{*}time+\beta 2^{*}intervention+\;\beta 3^{*}posttime+\varepsilon t \end{array}$$

multiple treatment periods analysis:

$$\begin{array}{r} Yt=\beta 0+\beta 1^{*}time+\beta 2^{*}intervention+\beta 3^{*}posttime\\ +\beta 4^{*}intervention2+\beta 5^{*}secondtime+\;\varepsilon t \end{array}$$

*Y*_*t*_ is the aggregated outcome variable measured at each equally spaced time point t (45), *time* is the time since the start of the study, *intervention* is a dummy (indicator) variable representing the intervention (preintervention periods 0, otherwise 1), *posttime* is the time after intervention number variable, *intervention 2* is the second intervention indicator variable, *secondtime* is the count change of the second intervention time. β0 represents the intercept and starting level of the outcome variable, β1 is the slope or trajectory of the outcome variable until the introduction of the prior intervention, β2 is the change in the level of the outcome that occurs in the period immediately following the introduction of the first intervention (compared with the preintervention period), β3 is the difference between preintervention and the prior intervention slopes of the outcome (46), β4 is the change in the level of the outcome that occurs in the period immediately following the introduction of the second intervention (compared with the prior intervention period), and β5 is the difference between the prior intervention and the second intervention slopes of the outcome. εt is the residual at time t, which represents the variation of the outcome variable not explained by the model (47).

We collected data of multiple interventions to estimate the post-intervention trends separately following the first and second policy intervention periods in the study. The first policy intervention point was the time at which the first or second batch of national-level pricing negotiations began. Gefitinib was included in the first batch of price-negotiated anti-cancer drugs in July 2016, and bevacizumab and recombinant human endostatin were included in the second batch in September 2017. As previously mentioned, Nanjing was included in January 2020 to expand the scope of the VBP pilot. Gefitinib (generic drug) was one of 25 centrally purchased drugs. To verify whether the VBP pilot program had a positive impact on the accessibility to gefitinib, we set the second policy intervention point of gefitinib (branded drug and generic drug) as January 2020. In addition, the second round of national-level pricing negotiations for bevacizumab and recombinant human endostatin were included in type B medicine list for national basic medical insurance from September 2017 to December 2019. These two drugs were once again covered by pricing negotiations and national basic medical insurance from January 2020 to December 2021(48). In general, further price reductions are expected during drug procurement renewals. Hence, we set January 2020 as the second policy intervention point for bevacizumab and recombinant human endostatin (Table 1).

The interrupted linear regression model requires that the outcome variable has a linear trend over time before and after the policy intervention and that the series has no autocorrelation. The Durbin-Watson (D-W) method was used to test for the existence of 1st order autocorrelation in the time series, with values close to 2 or 4 indicating no autocorrelation. The generalized least square estimator (GLSE) was used to correct any autocorrelation. The databases and plots were constructed using Excel 2020, and STATA v.16 software was used for statistical analysis. The test level was two-sided test α = 0.05.

## Results

### ITS analysis of changes in the trend of utilization

**Figure 2:**
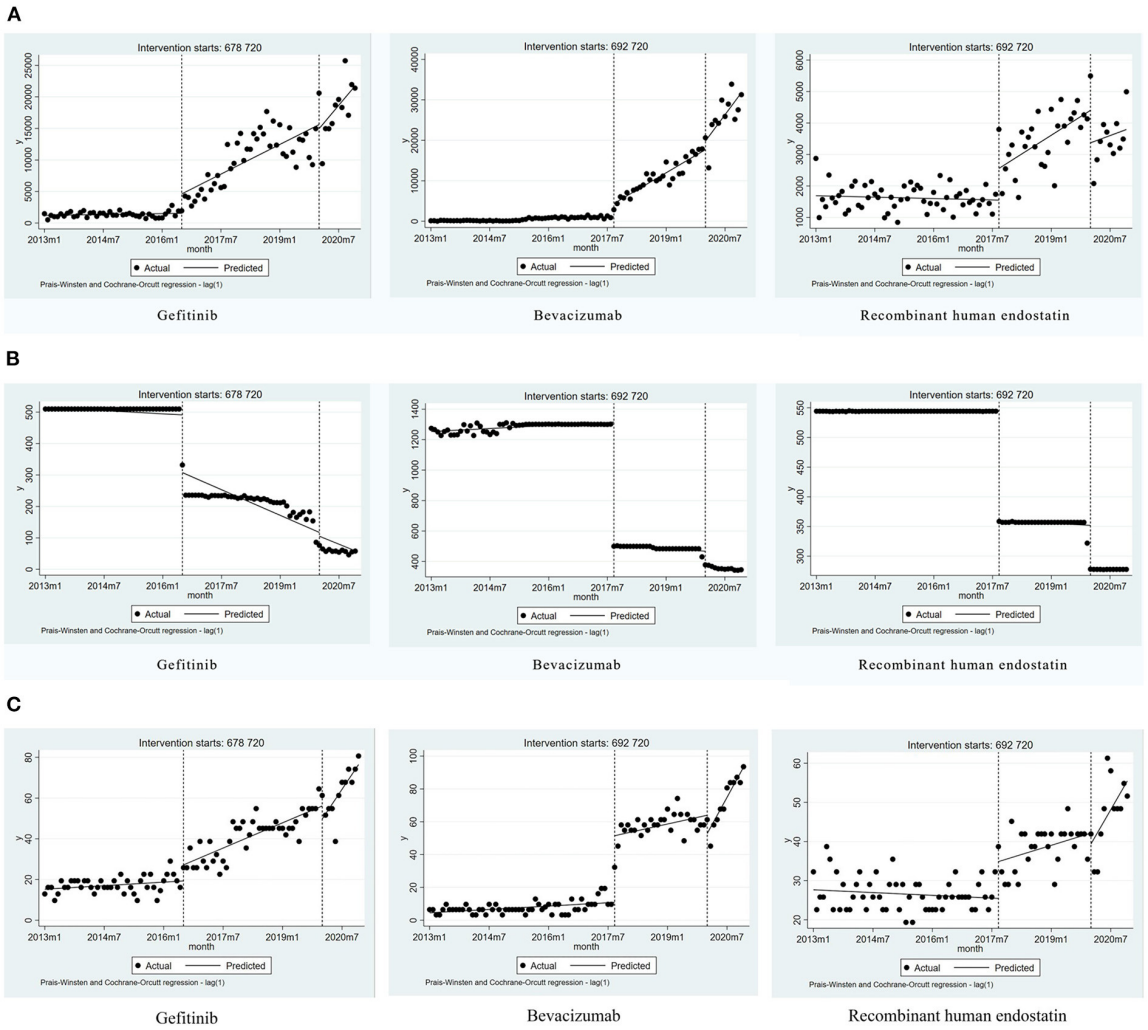
Results of the regression analysis of the monthly DDDs, the monthly DDDc, and the availability of study drugs before and after policy implementation. **(A)** The monthly DDDs of gefitinib, bevacizumab, and recombinant human endostatin. **(B)** The monthly DDDc of gefitinib, bevacizumab, and recombinant human endostatin. **(C)** The availability of gefitinib, bevacizumab, and recombinant human endostatin.

Gefitinib was included in the list of insured drugs in July 2016, and bevacizumab and recombinant human endostatin were included in September 2017. The time interval for all drugs was divided into two parts. The DDDs of all three drugs increased significantly after policy implementation (*P* < 0.001, *P* < 0.001, *P* = 0.008). The scatter plots of the observed monthly DDDs are shown in Figure 2. After setting January 2020 as the second policy intervention time, there was no significant difference between the monthly trend after the second intervention point and the monthly trend after the first intervention point for gefitinib (*P* = 0.416) and recombinant human endostatin (*P* = 0.750). However, the trend for bevacizumab increased by 397 per month after the second intervention point (*P* = 0.046, *95%CI* = 10.57, 1,209.81) (Table 2).

**Table 2:** Estimates from the multiple-treatment period's analysis of the impact of health policies on the monthly DDDs, the monthly DDDc, and the availability of the three medicines.

**Variable**	**DDDs**			**DDDc**			**Availability**		
	***β***	***P***	***95%CI***	***β***	***P***	***95%CI***	***β***	***P***	***95%CI***
**Gefitinib**									
Baseline level	1,212.15	0.000	898.95 to 1,525.25	511.60	0.000	508.69 to 514.52	15.23	0.000	12.90 to 17.56
Baseline trend	7.68	0.352	−8.64 to 24.00	−0.13	0.256	−0.35 to 0.09	0.10	0.126	−0.03 to 0.22
Level change	3,086.64	0.000	1,415.19 to 4,757.32	−241.34	0.000	−274.29 to −208.38	7.91	0.006	2.35 to 13.46
Trend change	253.64	0.000	173.87 to 333.41	−2.16	0.000	−3.08 to −1.23	0.59	0.000	0.37 to 0.82
The second level change	−647.57	0.848	−7,330.31 to 6,035.17	−94.37	0.000	−116.72 to −72.02	−6.37	0.282	−18.05 to 5.32
The second trend change	355.01	0.416	−509.00 to 1,219.13	−0.10	0.929	−2.44 to 2.23	1.74	0.009	0.44 to 3.03
**Bevacizumab**									
Baseline level	−100.65	0.039	−196.10 to −5.20	1252.86	0.000	1,236.90 to 1,268.82	4.51	0.000	2.97 to 6.04
Baseline trend	21.96	0.000	18.60 to 25.33	1.11	0.000	0.74 to 1.48	0.11	0.001	0.05 to 0.18
Level change	3,252.26	0.000	2,568.05 to 3,936.48	−807.23	0.000	−817.31 to −797.15	40.63	0.000	32.32 to 48.93
Trend change	453.68	0.000	403.75 to 503.61	−2.54	0.000	−3.52 to −1.57	0.33	0.152	−0.12 to 0.79
The second level change	2,128.29	0.331	−2,195.59 to 6,452.17	−90.07	0.000	−109.41 to −70.72	−11.15	0.063	−22.93 to 0.63
The second trend change	610.19	0.046	10.57 to 1,209.81	−2.17	0.026	−4.08 to −0.26	3.23	0.000	1.85 to 4.61
**Recombinant human endostatin**									
Baseline level	1,688.95	0.000	1,487.84 to 1,890.07	544.09	0.000	543.95 to 544.24	27.66	0.000	24.50 to 30.82
Baseline trend	−2.48	0.394	−8.22 to 3.27	0.00	0.241	−0.00 to 0.01	−0.04	0.386	−0.13 to 0.05
Level change	1,006.54	0.000	534.99 to 1,478.09	−184.65	0.000	−188.76 to −180.55	9.40	0.000	4.20 to 14.53
Trend change	68.38	0.000	42.79 to 93.97	−0.28	0.244	−0.74 to 0.19	0.30	0.035	0.02 to 0.58
The second level change	−1,031.83	0.080	−2,190.06 to 126.40	−74.41	0.000	−83.49 to −65.33	−2.79	0.523	−11.41 to 5.84
The second trend change	−27.40	0.750	−198.04 to 143.24	0.29	0.206	−0.15 to 0.75	1.20	0.018	0.21 to 2.20

### ITS analysis of changes in the daily cost

Due to the impact of the national pricing negotiations on targeted anti-cancer drugs, the monthly DDDc decreased significantly for gefitinib, bevacizumab, and recombinant human endostatin (*P* < 0.001, *P* < 0.001, *P* < 0.001). After setting January 2020 as the second policy intervention time, we found that the price reduction trend was not significantly different for gefitinib (*P* = 0.929) and recombinant human endostatin (*P* = 0.206). However, after the second policy implementation, there was a decrease in the trend for bevacizumab [*P* = 0.026, 95%CI = (−4.08, −0.26)] (Figure 2, Table 2). The price reduction rate was 71% for bevacizumab, 67% for gefitinib, and 49% for recombinant human endostatin.

### ITS analysis of changes in the availability

Eight secondary hospitals and 23 tertiary hospitals in Nanjing were included in the study. The availability of gefitinib, bevacizumab, and recombinant human endostatin increased significantly after the implementation of the NHIC policy (*P* < 0.001, *P* = 0.001, *P* < 0.001). The implementation of the second health policy was associated with a significant increase in the trend for gefitinib [*P* = 0.009, 95%*CI* = (0.44, 3.03)], bevacizumab [*P* < 0.001, 95% *CI* = (1.85, 4.61)] and recombinant human endostatin [*P* = 0.018, 95%*CI* = (0.21, 2.20)] (Figure 2, Table 2). The mean availability of these drugs before the national pricing negotiation was <30% in the surveyed hospitals, and increased to 60.33% after 2020, indicating that the drugs were available at many hospitals.

### Changes in affordability

**Figure 3:**
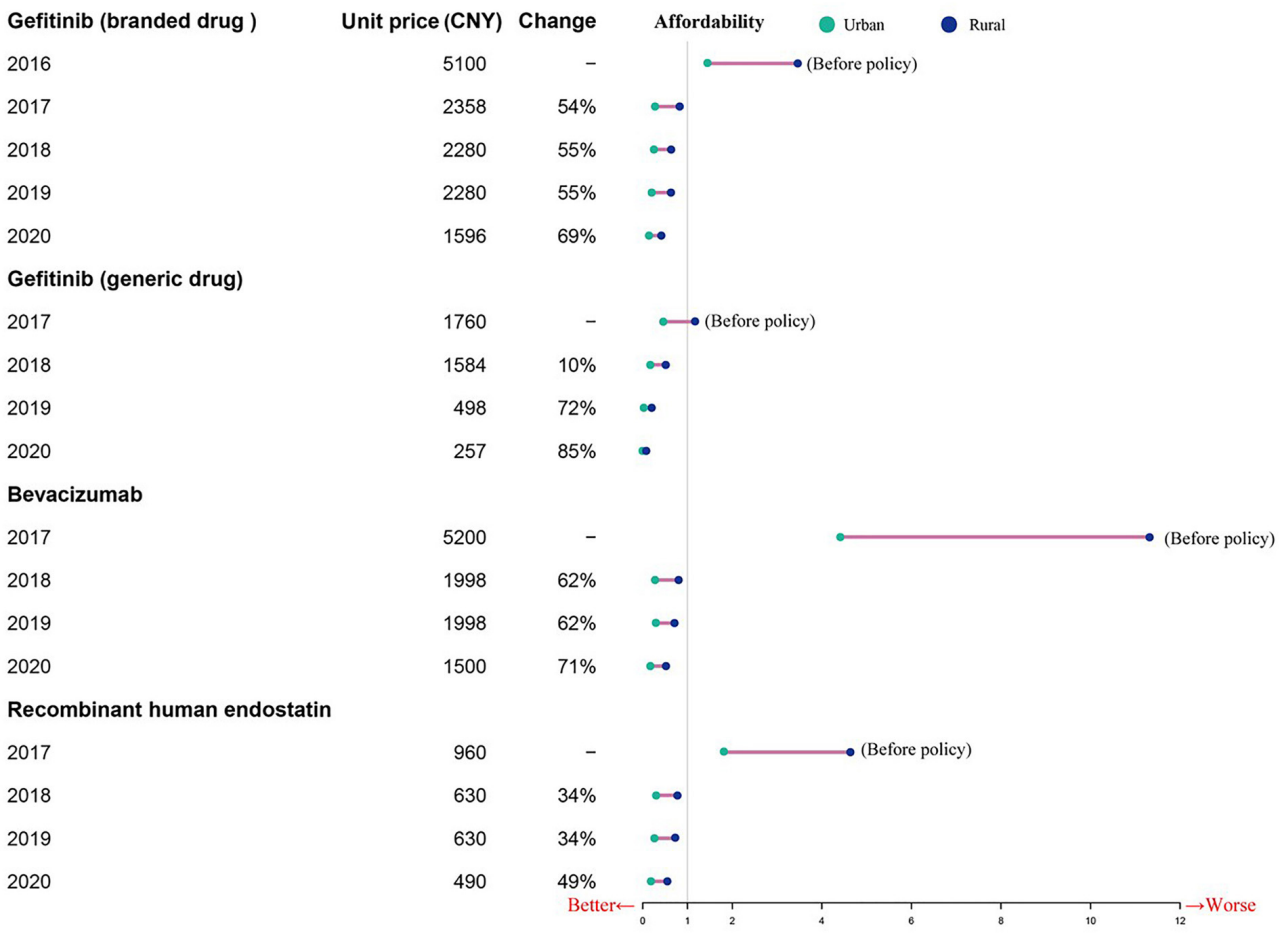
Change in affordability for mPFS treatment courses between urban areas and rural areas.

Before the NHIC policy implementation, only generic gefitinib was affordable to urban patients, and neither urban nor rural patients could afford the other targeted drugs. As shown in Table 3, the affordability of the drugs included in this study was 0.49 to 4.42 times the per capita annual disposable income for urban patients, and 1.16–11.31 times that for rural patients prior to policy implementation. After insurance coverage, the affordability was 0.2–0.33 times the per capita annual disposable income for urban patients, and 0.52–0.77 times that for rural patients in 2017. Due to the reduction of prices and the improvement of capita per-capita annual disposable income levels, the affordability of these drugs has increased every year after the implementation of the insurance policy (Table 3). The financial burden is higher for the rural patients compared to the urban patients, although the gap is narrowing (Figure 3).

**Table 3:** The change in patient affordability for mPFS treatment courses before and after the pricing negotiations.

**Variable**	**Specification**	**DDD (mg)**	**Treatment course**	**Year**	**Affordability before policy**		**Affordability after policy**			
			**based on mPFS (M)**		**Patient without PAP**	**Patient with PAP**				
Gefitinib (branded drug)	2,500 mg	250	4.6	2016	*U:*1.43; *R*:3.37	*U:*1.43; *R*:3.37				
				2017			*U:*0.30; *R*:0.77			
				2018				*U*:0.27; *R*:0.63		
				2019					*U*:0.25; *R*:0.58	
				2020						*U*:0.17; *R*:0.38
Gefitinib (generic drug)	2,500 mg	250	4.6	2017	*U:*0.49; *R:*1.16					
				2018			*U:*0.20; *R:*0.52			
				2019				*U:*0.06; *R:*0.14		
				2020					*U:*0.03; *R:*0.06	
Bevacizumab	100 mg/4 ml	25	6.1	2017	*U:*4.42; *R:*11.31	*U:*2.90; *R:*7.41				
				2018			*U:*0.31; *R:*0.73			
				2019				*U:*0.29; *R:*0.67		
				2020					*U:*0.21; *R:*0.47	
Recombinant human endostatin	15 mg/2.4 × 105 U/3 ml	8.5	6	2017	*U:*1.82; *R:*4.64					
				2018			*U:*0.33; *R:*0.77			
				2019				*U:*0.30; *R:*0.71		
				2020					*U:*0.22; *R:*0.51	

*The per capita annual disposable income:2016 CNY 49,998 in the urban area and CNY 21,156 in the rural area; in 2017 CNY 54,528 in the urban area and CNY 21,333 in the rural area; in 2018 CNY 59,308 in the urban area and CNY 25,263 in the rural area;2019 CNY 64,372 in the urban area and CNY 27,636 in the rural area;2020 CNY 67,553 in the urban area and CNY 29,621 in the rural area*.

*U, Urban; R, Rural; M, Months*.

## Discussion

### The utilization of targeted anti-cancer drugs has increased significantly

Given the high morbidity and mortality of lung cancer in China (49), the consumption of targeted drugs is substantial. We used an interrupted time-series design to conduct segmented regression analyses of the changes in the utilization of three targeted drugs commonly used to treat lung cancer. The national pricing negotiations and health insurance reimbursement policy and have significantly increased the utilization of gefitinib, bevacizumab, and recombinant human endostatin, and the trend was consistent throughout the observation period after the intervention (38). Multiple interventions were further used to estimate the post-intervention trends after the implementation of the national negotiation policy (50).

The monthly trends after the second intervention were not significantly different for gefitinib and recombinant human endostatin, indicating that the revised prices had a lasting positive impact on long-term utilization. However, the trend for bevacizumab was significantly different after the second intervention in January 2020. This change in the trend can be attributed to health insurance reimbursement and the renewal of negotiations in Nanjing, which further boosted its use. In addition, policy implementation resulted in an instantaneous upward effect, indicating that price is one of the key factors affecting the utilization of targeted anti-cancer drugs. Another possible explanation is that the risk associated with targeted anti-cancer drugs has reduced significantly, which is more conducive to clinical use. As the capital of Jiangsu province, the medical and health resources are mainly concentrated in Nanjing. In recent years, with improvement in traffic conditions, the medical institutions in Nanjing can provide health services for the patients from the surrounding cities or rural areas, such as some cities in Anhui province. It could lead to the steady increase in the volume of patient visits. According to the Nanjing Health Statistics Yearbook, from 2013 to 2020, the incidence of cancer patients with at least one hospital visit increased year by year. Therefore, the usage of three negotiated targeted anti-cancer medicines could be influenced by the number of patient visits during the study period. However, due to lack of control group in this study, we could not perform a qualitative analysis on the impact of patient visits.

### The price of the drugs included in this study has declined markedly

The cost of developing innovative anti-cancer drugs has soared in recent years. In order to recover costs and generate profits during the lifetime of the drug patent, pharmaceutical companies have to raise prices. To reduce the burden of health insurance and ensure significant profits for pharmaceutical companies, the government has launched the centralized strategic pricing negotiation policy through bulk sales. The national pricing negotiation policy and the VBP pilot program effectively lowered drug prices and relieved the economic pressure on the beneficiaries of insurance schemes.

Our study shows that the national pricing negotiation has successfully reduced the price of three negotiated targeted anti-cancer drugs. The daily cost of these drugs declined by over 49% after negotiation. A previous study showed that the prices of the three targeted drugs have dropped significantly after the first round of national drug pricing negotiation, with an average reduction of 58.6% (51). Three negotiations that were conducted from 2017 to 2019 for 150 drugs (including 57 targeted anti-cancer drugs), slashed the average daily cost by 54% (17). A nationwide study showed that compared with unregulated antineoplastics, the prices of regulated antineoplastic medications decreased after setting price caps (52). In the United States, Medicare is the most prominent financier for targeted anti-cancer drugs, followed by state Medicaid programs and commercial insurers (53). However, researchers at the University of Texas MD Anderson Cancer Center found that the high drug prices during and after their launch have contributed to increased spending (54). In addition, the price of new anti-cancer drugs has increased over time. Our results show that national pricing negotiation and health insurance reimbursement can successfully achieve OOP control.

In general, with the launch of generic drugs, the price and accessibility of branded drugs may decrease. According to the database of the Nanjing Regional Hospital Drug Analysis System, domestic generic gefitinib and bevacizumab launched in February 2017 and March 2020, respectively, while there was no generic drug for recombinant human endostatin. Our data was retrieved from January 2013 to December 2020. Therefore, whether the launch of generic drugs affects the price and accessibility, our study mainly focuses on the impact of generic gefitinib on branded gefitinib. From Figure 2, we can intuitively see that after generic gefitinib launched in February 2017, the trend of the monthly DDDs, the monthly DDDc, and the availability did not change much, and basically remained at the same level. Therefore, it is temporarily impossible to conclude the impact of the launching of generic drugs on the price and accessibility of branded drugs from the data in this paper.

### Positive effects of pricing negotiation, the VBP pilot program and the NHIC policy on drug availability

Implementing the NHIC policy is an important step in guiding the procurement and availability of essential anti-cancer drugs for the public sector. Our findings indicate that the mean availability of the targeted anti-cancer drugs was <30% in Nanjing City prior to the national pricing negotiations. The drugs were available at few hospitals, which were only at full cost as an OOP expense and unavailable in the rest hospitals due to unreliable supply. However, the availability of these drugs increased significantly to 60.33% in 2020 after the implementation of the aforementioned policies and has shown sustained growth in the long term. Some studies have reported greater availability of anti-cancer drugs in private hospitals (71%) compared to public hospitals (43%) (55, 56). Possible reasons for the low availability 7 of drugs in public hospitals include inaccurate estimation of the demand, poorly managed supply chain systems, an underfunded public health sector, or lack of commercial motivation (57). Thus, pricing negotiation can help control pharmaceutical spending for hospitals, which highlights the need to streamline drug procurement, distribution, and supply.

### The affordability of patients has improved

Anti-cancer treatments were not affordable for most families, which often led to treatment abandonment (5–8, 54). A retrospective observational study focusing on the utilization of targeted therapies in Taiwan showed that targeted therapies were representing a substantial economic burden (58). The number of days a daily wage worker would have to work to afford anti-cancer treatment depends on the treatment protocol, indications, and the economic output per person. There are significant differences in the affordability of anti-cancer drugs worldwide. Based on individual income, the patients in the low-and middle-income countries have lower affordability compared to high-income countries (59, 60).

We found that the affordability of the three anti-cancer drugs has increased every year after the implementation of the aforementioned policies in Nanjing, although there are still considerable differences between urban and rural areas. And, other studies on the price negotiation system of special medical insurance drugs in 6 typical provinces in China found similar positive effects on affordability of expensive targeted anti-cancer drugs (38, 61). The financial burden of rural patients is higher than that of urban patients, although the gap is narrowing. These differences were driven by national drug pricing negotiations, centralizing procurement, the gap in per capita annual disposable income and lower ratios of individual payment needed after the implementation of NHIC policy. Moreover, the affordability of individual patients is transient since multiple clinical examinations, standard tests, and chemotherapy over a long period incur high total costs. Therefore, a supplementary measure should be in place to top up the basic cover offered by the basic social health insurance schemes.

Pricing negotiations, centralizing procurement, and implementation of the NHIC policy can promote the utilization and affordability of anti-cancer drugs. This indicates that the cost of anti-cancer treatments and the affordability of individual patients were the major factors contributing to the inequity. Therefore, the cost and affordability should be taken into consideration when negotiating medicine procurement terms. Consistent with other previous findings, the barriers to the accessibility of negotiated targeted anti-cancer drugs include high prices, limited coverage of public insurance schemes (62), inequality across insurance schemes, regional variations (59), non-availability of the medicine at the facilities, and updated clinical diagnosis and treatment standards (63). This in turn could be due to the differences in the high cost of anti-cancer drugs, the burden of disease, disease priorities, the capacity of the health insurance system, government budget management, regional economic development, and unequal diagnosis and treatment capacities of medical institutions (64, 65).

To the best of our knowledge, this is the first study to measure the accessibility of anti-cancer drugs over an 8-year period after national health policy implementation. We analyzed 96 months of data before and after the policy was implemented to comprehensively assess the long-term influence of government health policy. Furthermore, ITS analysis for single and multiple treatment periods was used to compare the trends in utilization, price, and availability of anti-cancer drugs. We demonstrated the impact of national health policy based on multiple interventions, by estimating post-intervention trends separately following pricing negotiations, NHIC policy and the VBP pilot program. Nonetheless, there were a few limitations in the study. Due to limited data access, only one city was included in our study, and the results may not be generalized to the other regions of China, especially for backward areas. As far as we know, the social security agency of Nanjing had put kinds of negotiated drugs into the scope of Special Medicine Management System since 2017. The management model of medical institutions, responsible physicians, retail pharmacies and infusion centers was implemented in Nanjing city. Gefitinib and recombinant human endostatin were included in the scope of Special Medicine Management System in 2017, and bevacizumab was included in 2018 (66). In addition, private hospitals and retail pharmacies were not included in our study. This could indeed have an impact on the accessibility of drugs in the hospital channel. The inclusion of more purchasing data from different medical institutions may help reduce the selection bias to a certain extent.

## Conclusion

Trends in the accessibility of targeted anti-cancer drugs increased significantly after the implementation of the national pricing negotiation, the NHIC policy and the VBP pilot program and showed sustained long-term growth. Lower drug prices relieve the economic pressure on the beneficiaries of the insurance schemes and achieve OOP control. However, the further study aims to generate evidence to inform the government health coverage of negotiated targeted anti-cancer medicines as a more inclusive and equal policy, through each of the needed patients can get access to the anti-cancer medicines regardless of regional variations, types of cancer, or the ability to pay. In the future, multi-pronged supplementary measures and policy approaches by multiple stakeholders (government, financiers, and pharmaceutical companies) such as national price negotiation, PAPs, efficient resource allocation, issuance of compulsory licenses for procurement, and other special marketing arrangements will facilitate equitable access and use of effective and affordable innovative anti-cancer drugs.

## Data availability statement

The raw data supporting the conclusions of this article will be made available by the authors, without undue reservation.

## Author contributions

XL and YL conceptualized and designed the whole study. YL and HY were responsible for collecting and analyzing data. YL drafted the initial manuscript. KF took the responsibility for editing. All authors contributed to the critical revision of the manuscript and approved the final version.

## Funding

This work was supported by the National Natural Science Foundation of China (72074123 and 71673147) and the China Medical Board (Grant No: 17-277).

## Conflict of interest

The authors declare that the research was conducted in the absence of any commercial or financial relationships that could be construed as a potential conflict of interest.

## Publisher's note

All claims expressed in this article are solely those of the authors and do not necessarily represent those of their affiliated organizations, or those of the publisher, the editors and the reviewers. Any product that may be evaluated in this article, or claim that may be made by its manufacturer, is not guaranteed or endorsed by the publisher.

## Authors disclaimer

The contents are solely the responsibility of the authors and do not reflect the views of the funding bodies or any organization.
